# Current molecular biomarkers evaluation in gastric/gastroesophageal junction adenocarcinoma: pathologist does matter

**DOI:** 10.1007/s13304-022-01330-5

**Published:** 2022-07-14

**Authors:** Gianluca Businello, Valentina Angerilli, Sara Lonardi, Francesca Bergamo, Michele Valmasoni, Fabio Farinati, Edoardo Savarino, Gaya Spolverato, Matteo Fassan

**Affiliations:** 1 Department of Medicine (DIMED), University of Padua, Padua, Italy; 2 Veneto Institute of Oncology, Istituto Di Ricovero E Cura a Carattere Scientifico (IRCCS), Padua, Italy; 3 Department of Surgery, Oncology and Gastroenterology (DISCOG), University of Padua, Padua, Italy

**Keywords:** Gastric adenocarcinoma, Gastroesophageal junction adenocarcinoma, Biomarkers, Immunohistochemistry

## Abstract

The comprehensive molecular characterization of gastric and gastroesophageal junction adenocarcinomas has led to the improvement of targeted and more effective treatments. As a result, several biomarkers have been introduced into clinical practice and the implementation of innovative diagnostic tools is under study. Such assessments are mainly based on the evaluation of limited biopsy material in clinical practice. In this setting, the pathologist represents a key player in the selection of patients facilitating precision medicine approaches.

## Introduction

Gastric cancer (GC) represents a major health issue worldwide. With over one million new cases in 2020 and 769,000 related deaths, GC ranks globally fifth for incidence and fourth for mortality among malignancies [1]. Incidence and mortality vary across different regions and ethnicities. GC is the most commonly diagnosed malignant neoplasm and the first cause of cancer-related death in several South-Central Asian countries. Moreover, Eastern Asia and Eastern Europe have the highest GC incidence rates, while Northern America and Northern Europe have the lowest [1]. It is also noteworthy that a decrease in distal GC incidence has been observed during the last 50 years in Western countries. This trend may be explained by the decreased prevalence of Helicobacter Pylori infections and the improvement of health and hygiene standards [2]. On the other hand, a higher incidence of proximal GC (*i.e.* of gastric cardia and gastroesophageal junction) has been reported [3], probably related to the increasing incidence of gastroesophageal reflux disease and obesity [4].

## Histological and molecular classification of gastric cancer

GC is a heterogeneous neoplasm from both a phenotypical and a molecular viewpoint. In the last 60 years, many histopathological and, more recently, molecular classifications have tried to dissect the biological complexity of GC. Molecular characterizations of GC also allowed to set the stage for the development of molecular targeted therapies and the discovery of predictive biomarkers.

Gastric/gastroesophageal junction adenocarcinoma (GAC) accounts for more than 90% of GCs, representing the most common histotype among gastric malignancies. Other types of GC are squamous cell carcinoma, adenosquamous carcinoma, undifferentiated carcinoma, gastroblastoma and neuroendocrine neoplasms [5].

Among GAC’s morphological classifications, the WHO and Laurén’s are two of the most widely used.

2019 WHO classification recognizes five main subtypes of GAC: (i) tubular adenocarcinoma, (ii) papillary adenocarcinoma, (iii) mucinous adenocarcinoma, (iv) poorly cohesive carcinoma (PCC) and (v) mixed adenocarcinoma. Other rarer variants are GAC with lymphoid stroma, hepatoid carcinoma, micropapillary carcinoma and GAC of fundic-gland type [5].

Laurén’s classification subdivides GAC into intestinal (53%), diffuse (33%) and indeterminate (14%) types [6]. Despite having a lesser degree of sophistication than the WHO classification, Lauren’s classification effectively emphasizes crucial aspects of GAC and is commonly used by clinicians and surgeons. In fact, the intestinal and diffuse subtypes present not only different morphologic appearances but also different molecular background: intestinal-type GAC is usually associated with Helicobacter Pylori and Correa’s cascade, while the diffuse type is characterized by loss of E-cadherin expression [7].

However, in the era of personalized oncology, the morphology-based evaluation must be integrated with additional molecular biomarkers assessment. In this context, two NGS-based molecular classifications have been introduced: The Cancer Genome Atlas (TCGA; 2014) and the Asian Cancer Research Group (ACRG; 2015).

According to TCGA classification, GAC may be subdivided into: (i) tumors positive for Epstein-Barr virus (EBV) (9%), (ii) microsatellite instability-high (MSI) tumors (21%), (iii) genomically stable (GS) tumors (20%) and (iv) tumors with chromosomal instability (CIN) (50%) [8].

EBV-positive GACs commonly arise in the gastric body or fundus and usually present high levels of DNA promoter hypermethylation and of CpC island methylation (CIMP). Specific molecular alterations of these tumors are mutations of *PIK3CA* (80%), *ARID1A* (55%), and *BCOR* (23%), together with *CDKN2A* promoter hypermethylation (100%) [8]. Moreover, overexpression of Programmed Death Ligands 1 and 2 (PD-L1 and PD-L2) and significant lymphoid infiltration are commonly found in EBV-positive GACs [9].

MSI GACs are defined by loss of mismatch repair (MMR) functions, usually related to hypermethylation of *MLH1* promoter region [10]. These neoplasms typically occur in the gastric antrum of older patients, but they may arise in the context of Lynch syndrome and non-polyposis colorectal cancer syndrome [11]. The majority of MSI GACs show intestinal-type morphology and have been associated with a lower rate of nodal metastasis and a more favorable prognosis [12].

GS GACs usually develop in the distal stomach of younger patients (median age 59). Usually associated with Laurén’s diffuse histotype, GS tumors have the worst overall survival and prognosis among TCGA subtypes [8]. This group is characterized by low copy number alterations and a low mutation rate, and the most common mutated genes are *ARID1*, *RHOA*, and *CDH1*. In about 15% of GS GACs a fusion between *CLDN18* and *ARHGAP26* does occur, which is mutually exclusive with *RHOA* mutations [8].

CIN GACs usually arise at the gastroesophageal junction and commonly present an intestinal histotype [13]. Characteristic of these neoplasms are DNA aneuploidy, highly variable chromosomal copy numbers, and mutations of *TP53*, with consequent chromosomal instability. Moreover, genomic amplifications of *EGFR*, *ERBB2*, *ERBB3*, *MET*, *FGFR2*, and *VEGF-A*, are frequent [8].

ACRG classification proposes four other molecular subtypes of GACs, *i.e.*: (i) microsatellite unstable (MSI) tumors (23%); (ii) microsatellite stable with epithelial-mesenchymal transition features (MSS/EMT) tumors (15%); (iii) microsatellite stable with *TP53* active (MSS/TP53^+^) tumors (26%); and iv) microsatellite stable with *TP53* inactive (MSS/ TP53^−^) tumors (36%) [14].

MSI GACs usually have an antral location and intestinal histotype. Often diagnosed at an early stage, MSI GAC is characterized by the best prognosis among ACRG subtypes. Hypermutation, loss of *MLH1* expression and elevated DNA methylation signature are characteristic of this subtype. Commonly mutated genes in MSI GACs are *ARID1A* (44.2%), PI3K-PTEN-mTOR pathway-related genes (42%), *KRAS* (23.3%), and *ALK* (16.3%) [14].

MSS/EMT GACs are associated with an earlier onset and with diffuse histotype. MSS/EMT GC is the ACRG subtype with the worst prognosis and is associated with advanced stage at diagnosis, a high percentage of recurrence and a high frequency of peritoneal metastases. This subgroup is characterized by loss of E-cadherin expression and by a low tumoral mutational burden [14].

MSS/TP53^+^ GACs are associated with intestinal histotype, EBV infection and a high percentage of liver-limited metastasis. Mutations in *ARID1A*, *PIK3CA*, *SMAD4*, *KRAS*, and *APC* are commonly reported [14].

MSS/TP53^−^ tumors usually present an intestinal histotype and are more common in males. Among ACRG subtypes, MSS/TP53^−^ GACs have the highest prevalence of *TP53* mutations (60%). Moreover, recurrent amplification of *ERBB2*, *EGFR*, *CCNE1*, *CCND1*, *MDM2*, *ROBO2*, and *GATA6* have been described [14].

Despite every proposed classification that shed light on different aspects of GC, a flawless and unifying classification is still lacking [15]. More efforts are required to dissect GC heterogeneity to develop more efficient predictive biomarkers and targeted therapies.

Through the analysis of large molecular data from different platforms, the landmark study by TCGA in 2017 demonstrated that GEJ cancers and GCs have notable molecular similarities, with a progressive increase of chromosomal instability phenotype proximally [16]. Although GEJ cancer has been considered separate from GC according to a model whereby EAC originates from Barrett’s esophagus and thus is not of gastric origin, this growing body of evidence indicates that they should be considered as a single entity for clinical trials of neoadjuvant, adjuvant or systemic therapies.

## Targeted therapies in clinical practice

**Fig. 1:**
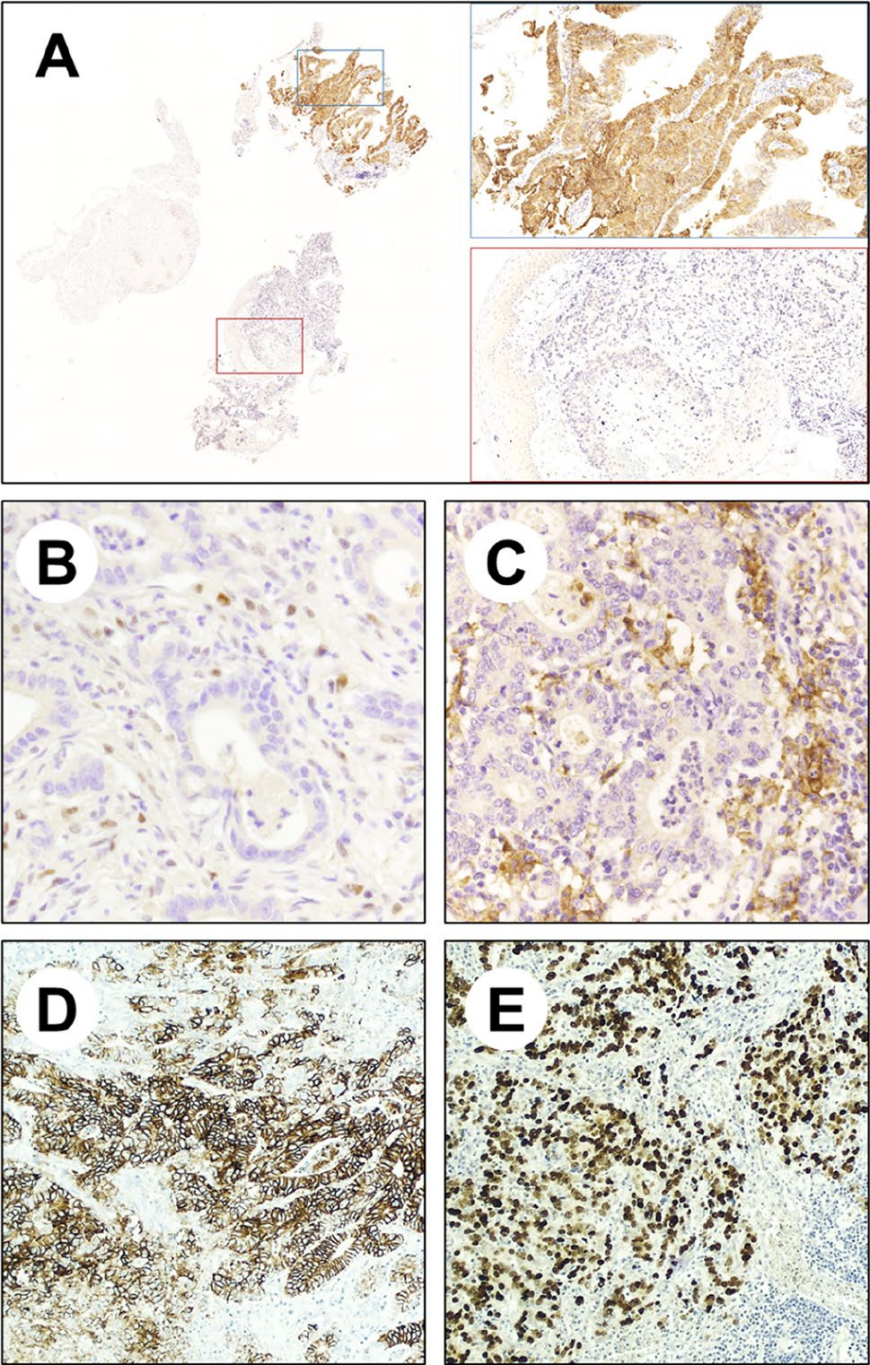
Immunohistochemical evaluation of predictive biomarkers in gastric/gastroesophageal adenocarcinoma. **A** Heterogeneous Her2 expression in a gastroesophageal junction adenocarcinoma showing the concomitant presence of a 3^+^ positive and a negative biopsy. **B** Loss of the mismatch repair complex (MMR) protein PMS2. **C** PD-L1 Combined Positive Score (CPS) > 10. **D** Membranous expression of CLDN18. (E) EBER (Epstein–Barr virus-encoded small RNAs) in situ hybridization in an EBV-associated adenocarcinoma

The scientific and medical community is making great efforts toward the development of new molecular targeted therapies (Table 1). At present, the use of anti-Her2 targeted therapies and immune check-point inhibitors (ICIs) is well-established in the clinical setting (Fig. 1). Other promising/appealing molecular targeted therapies are currently being evaluated in clinical trials. In high-incidence countries, such as Japan and Korea, multiple biomarkers, including Her2, MMR proteins, EBV, PD-L1, EGFR, FGFR2, and CLDN18.2, are routinely evaluated by means of immunohistochemistry or in situ hybridization in all GC patients [17]. In lower-incidence Western countries, Her2 and PD-L1 assessment on biopsy samples should be mandatory before initiation of the first-line treatment in all patients with GC. The immunohistochemical assessment of the MMR proteins and EBV status is recommended, according to tissue availability.

**Table 1:** Selected clinical trials involving molecular-targeted therapies in Gastric and GEJ cancers

Molecular target	Therapeutic agent	Trial	Tumor type	Treatment	Line of therapy	Outcomes
HER2	Trastuzumab	ToGA [22]	Her2 3 + and FISH- positive Gastric and GEJ cancers	Capecitabine or 5-FU plus cisplatin with trastuzumab *vs* capecitabine or 5-FU plus cisplatin without trastuzumab	First line	ORR: 47% vs 35%; OS: 13.8 vs 11.1 months; PFS: 6.7 vs 5.5 months
	Trastuzumab-Deruxtecan (T-Dx)	DESTINY-Gastric 01 [42]	Her2 3 + , Her2 2 + and ISH-positive Gastric cancers	T-Dx *vs* irinotecan or paclitaxel	Third or later line	ORR: 51% vs 14%; OS: 12.5 vs 8.4 months; PFS: 5.6 vs 3.5 months
PD-1	Nivolumab	CHECKMATE-649 [58]	Unselected Gastric and GEJ cancers	Capecitabine plus oxaliplatin or FOLFOX with nivolumab *vs* capecitabine plus oxaliplatin or FOLFOX without	First line	ORR: 60% vs 45%; OS: 14.4 vs 11.1 months; PFS: 7.7 vs 6.1 months
CLAUDIN 18.2	Zolbetuximab	FAST [74]	CLDN18.2 2 + and 3 + (≥ 40% tumour cells) Gastric and GEJ cancers	Capecitabine plus oxaliplatin and epirubicin with zolbetuximab *vs* capecitabine plus oxaliplatin and epirubicin without zolbetuximab	First line	ORR: 49.4% vs 33.3%; OS: 13.0 vs 8.4 months; PFS: 7.5 vs 5.3 months
FGFR2	Bemarituzumab	FIGHT [92]	FGFR2b + or FGFR2 amplified Gastric and GEJ	Modified FOLFOX6 with bemarituzumab *vs* modified FOLFOX6 without bemarituzumab	First line	ORR: 47% vs 33%; OS: not reached vs 12.9 months; PFS: 9.5 vs 7.4 months
VEGFR	Ramucirumab	RAINBOW [102]	Unselected Gastric and GEJ cancers	Paclitaxel with ramucirumab *vs* paclitaxel without ramucirumab	Second line	ORR: 28% vs 16%; OS: 9.6 vs 7.4 months; PFS: 4.4 vs 2.9 months

*GEJ* gastroesophageal junction, *ORR* objective response rate, *OS* overall survival, *PFS* progression-free survival)

### Anti-Her2 targeted therapy

The member of the epidermal growth factor receptor (EGFR) family, Her2 or ERBB2 (erb-b2 receptor tyrosine kinase 2) is involved in transmembrane signaling, and its overexpression is responsible for increased cell proliferation, growth, and cell survival [18]. Her2 overexpression has been associated with intestinal histotype and proximal GACs, being reported up to 30% of gastroesophageal junction’s cancers [19, 20]. Assessment of Her2 status is based on immunohistochemistry (score 0, 1^+^, 2^+^, and 3^+^); for tumors with Her2 score 2^+^, fluorescent in situ hybridization (FISH) should be performed [21]. Her2 -positive tumors are defined by a Her2 score 3^+^ or by a Her2 score 2^+^ with FISH-detected *HER2* amplification [20].

Following the results of the ToGa trial, tumor biopsies from all patients with GAC at an advanced stage should be assessed for Her2 status [22]. In fact, in patients with advanced Her2-positive GAC, a combination of trastuzumab and fluoropyrimidine plus cisplatin chemotherapy significantly improves overall survival (OS: 13.8 vs 11.1 months) [22]. Care must be taken in the assessment of Her2 status, since preinvasive lesions may also harbor *HER2* amplifications and overexpression [23]. Moreover, Her2 expression may be extremely heterogenous in GAC, even within individual neoplasms, with a great impact on trastuzumab efficacy. A heterogeneous expression of Her2 in primary GAC has been associated with shorter progression-free survival (PFS) after trastuzumab-containing first-line chemotherapies [24–26]. Therefore, it is highly recommended to acquire tumoral material representative of the entire lesion, performing at least 5 biopsies [27]. Moreover, 1–14% of GACs also present a discordant Her2 expression between primary and metastatic tumors [28–34]. According to the GASTHER1 study [35], patients with Her2 positive disease identified in repeated biopsies of recurrent and/or metastatic lesions in the context of initially Her2 negative primary GAC, have PFS comparable to those with Her2 positive disease on initial assessment, after trastuzumab-containing chemotherapy. Hence, it is recommended to repeat the Her2 status assessment in primary and metastatic lesions in patients with advanced GAC and initially Her2 negative primary neoplasm.

Primary resistance to trastuzumab is reported in about 50% of GAC patients treated with combined therapy [22]. It has been suggested this phenomenon may be linked to alterations in RTK-RAS-PI3K/AKT pathway and related genes (*e.g., EGFR*, *FGFR2*, *MET*, and *KRAS*) [8, 36, 37].

The effectiveness of trastuzumab in responder patients may also be compromised by the development of acquired resistance, the causes of which have not been completely elucidated yet [9]. Heterogeneity of Her2 expression can partially explain this phenomenon: trastuzumab-containing chemotherapies, while eradicating Her2 positive neoplastic clones, also confer a proliferative advantage to Her2 negative malignant cells [38, 39]. Moreover, loss of *ERBB2* amplification, focal *ERBB2* exon 16 deletion and alterations in the RTK/RAS/PI3K pathway have been reported to occur in GAC patients after trastuzumab-containing chemotherapy [37]. In a study by Kim and colleagues [40], *CCNE1* amplifications have been linked to a lower response rate to lapatinib combined with capecitabine and oxaliplatin as first-line neoadjuvant therapy in Her2 positive GAC patients. In the same study, analysis of circulating tumor DNA (ctDNA) revealed the progressive onset of *MYC*, *EGFR*, *FGFR2*, and *MET* amplifications in the progression phase of the disease.

To overcome the issue of primary and acquired resistance to trastuzumab, new Her2-targeted molecules and combinations have been developed.

The antibody–drug conjugate trastuzumab-deruxtecan (T-Dx) is composed of a molecule of trastuzumab attached to a cytotoxic topoisomerase I inhibitor by a cleavable tetrapeptide-based linker. This drug is able to affect not only Her2 positive cells but also the surrounding Her2 negative neoplastic clones [41]. According to Shitara and colleagues [42], T-Dx significantly improves OS (12.5 vs 8.4 months) in patients with Her2 positive advanced-stage GACs previously treated with at least two lines of therapy, including trastuzumab. Of note, a significant antitumoral activity of T-Dx has also been reported in patients with Her2-low GACs (*i.e.* GACs with Her2 score 2^+^ and FISH negative) [43]. Ongoing clinical trials (NCT03821233, NCT03602079 and NCT03255070) are trying to evaluate the efficacy of other antibody-drug conjugates against Her2-positive GACs [20].

A synergistic antineoplastic activity between anti-Her2 and anti-PD-1 agents has been reported [44]. This effect could be explained because anti-Her2 drugs may induce PD-1/PD-L1 expression, increase tumor-infiltrating immune cells and promote cross-presentation by dendritic cells [45, 46]. These observations have represented the rationale for several clinical trials. Results from a randomized phase III trial (NCT03615326) support the efficacy of pembrolizumab plus trastuzumab-including chemotherapy in Her2 positive GACs, with a significant reduction in tumor size and improvement in objective response rate (ORR); complete responses in some participants have also been reported [47]. A combination of the anti-Her2 antibody margetuximab and pembrolizumab is also being under evaluation in patients with previously treated, Her2 positive GACs [48]. Moreover, the phase II/III MAHOGANY trial [49] is currently evaluating margetuximab plus retifanlimab with or without chemotherapy and margetuximab plus tebotelimab with chemotherapy in first-line unresectable metastatic or locally advanced GACs. A combination of T-Dx plus the anti-PD-1 antibody is currently under evaluation in phase I/II trial NCT04379596 [50].

Other new anti-HER2 molecules include tucatinib and ZW25, and their efficacy in the treatment of Her2-positive GC is currently under investigation [20, 51].

It has to be noted that Her2 immunohistochemical evaluation still remains a superior predictive biomarker than more sophisticated next-generation technologies. This further underlines the role of the pathologist’s expertise in the therapeutic stratification of GAC patients.

### Immunotherapy

ICIs act by reducing neoplastic immune tolerance. ICIs include antibodies against PD1 (pembrolizumab, nivolumab), PD-L1 (atezolizumab, avelumab, durvalumab) and CTLA-4 (ipilimumab) [52].

Potential biomarkers for response to ICIs include PD-L1 expression, microsatellite instability, positive EBV status and high tumor mutational burden (TMB) [53]. PD-L1 expression in GC is determined immunohistochemically by using Combined Positive Score (CPS), which is defined as the number of PD-L1 positive tumor cells, macrophages and lymphocytes, divided by the total number of neoplastic cells, multiplied by 100. GAC is considered PD-L1 positive if CPS ≥ 1.

It must be noted that only invasive neoplasia must be assessed for PD-L1 expression: dysplasia (formerly known as intraepithelial neoplasia) must not be considered when assessing immunohistochemical biomarkers. In fact, PD-L1 has also been described in gastric dysplastic lesions [7, 54]. Of note, our group [55] first described a case of an EBV-associated foveolar gastric dysplasia, which may represent another rare but possible pitfall in the assessment of patients’ eligibility for immunotherapy.

ICIs have been approved in the treatment of selected patients with advanced GAC, but with limited benefit. Pembrolizumab has been approved in the USA as third-line (or greater) therapy in patients with GACs with CPS score ≥ 1 [56]. Nivolumab is used in Japan as third-line option to treat patients with advanced GACs disregarding PD-L1 status [57]. In the phase III CheckMate-649 trial, first-line treatment with nivolumab plus chemotherapy has shown significantly improved survival benefit in previously untreated patients with PD- L1 positive advanced GAC, when compared to chemotherapy alone (OS: 14.4 vs 11.1 months) [58].

On the other hand, some randomized trials have failed to demonstrate the superiority of ICIs above chemotherapy in terms of clinical advantages. In the KEYNOTE-061 study, pembrolizumab has not significantly improved OS compared with paclitaxel as second-line therapy for advanced GACs with PD-L1 CPS score ≥ 1 [59]. According to the KEYNOTE-062 trial’s results, pembrolizumab alone or pembrolizumab plus chemotherapy were not superior to chemotherapy in terms of OS and PFS [60].

Loss of MMR proteins’ function is mostly caused by mutational inactivation or epigenetic silencing of DNA MMR genes, resulting in microsatellite instability. In clinical practice, microsatellite status is evaluated by molecular assays or, as a surrogate, by immunohistochemical analysis of the expression of MMR proteins (MLH1, PMS2, MSH2 and MSH6) [10]. FDA approved the use of pembrolizumab as second-line therapy against MSI-GC [61], but it is worth remembering the majority of MSI-GC are localized and only 3–4% of metastatic GACs are MSI [56, 59].

Amplification of genes encoding for PD-L1 (*CD274*) and PD-L2 (*PDCD1LG2*) have been described in EBV-positive GCs [8], suggesting the use of ICIs in this molecular subtype. In a study by Kim and colleagues [62], a significant response to pembrolizumab as a second or greater line of therapy in patients with EBV-positive GCs has been observed (ORR of 100%). In the same study, an ORR of 85.7% in MSI-GCs has also been reported.

TMB may represent a novel predictive biomarker for ICIs, but its efficacy has not been established yet, and further studies are needed. Kim and colleagues have investigated the value of TMB as a potential biomarker of response to pembrolizumab in advanced GAC [62]. However, results were unsatisfactory: high TMB (*i.e.,* > 10–15 mutations per megabase according to different adopted sequencing panels) was associated with MSI subtype, but not all patients with high TMB attained an objective response. Conversely, according to the results of the phase II KEYNOTE-158 study [63], high TMB may identify a subgroup of GAC patients who could significantly respond to pembrolizumab monotherapy. Finally, Greally and colleagues [64] reported an association between TMB and improved survival only in MSI GAC patients.

Other possible ICIs-based therapies are currently under investigation. Results from early phase trials have suggested that combinations of anti-PD-1 antibody plus tyrosine kinase inhibitors (TKIs) may be effective against GAC. Reported ORRs have, respectively, been 44% for regorafenib plus nivolumab [65], and 69% of lenvatinib plus pembrolizumab [66].

In phase I/II NivoRam trials [67], a combination of nivolumab plus the anti-VEGFR-2 ramucirumab in second-line treatment for advanced GC has been evaluated, with promising results. Finally, a combination of nivolumab and ipilimumab is currently being studied in patients with GAC [53].

## Future directions

### Anti-Claudin 18.2 therapies

Claudin 18.2 (CLDN18.2) is a component of intercellular junctions specific for gastric epithelial cells and is expressed in a significant percentage of primitive and metastatic GACs [68, 69]. As discussed above, up to 15% of GS GACs harbor *CLDN18–ARHGAP26/6* fusions [8], and the presence of the fusion is related to CLDN18.2 expression in almost all cases [70]. Expression of CLDN18.2 is extremely heterogenous in GC, and 6 biopsies should be considered as a standard of sampling for an adequate immunohistochemical profiling [71]. Since CLDN18.2 is a trans-membranous protein, it represents an ideal target for monoclonal antibodies [68]. Zolbetuximab is a recently developed antibody that binds CLDN18.2 with consequent antibody-dependent and complement-dependent cell death [72]. Phase IIa MONO trial reports an ORR of 9% and a disease control rate of 23% using zolbetuximab as a single agent in patients with recurrent or refractory advanced GACs [73]. In phase II study FAST, the combination of zolbetuximab and EOX (epirubicin, oxaliplatin and capecitabine) as first-line chemotherapy significantly improves PFS (7.5 vs 5.3 months) and OS (13.0 vs 8.4 months) in patients with CLDN18.2 positive GACs [74]. Moreover, phase III studies NCT03504397 and NCT03653507 are currently evaluating the addition of zolbetuximab to first-line chemotherapy in GACs with high levels of CLDN18.2 expression [20].

Finally, the use of CLDN18.2-targeted chimeric antigen receptor (CAR) T against CLDN18.2 positive gastric PADX models has been investigated, with preliminary but encouraging results [20, 75].

### PARP inhibitors

About 10% of GACs harbor mutations in genes involved in homologous DNA repair (*e.g. BRCA1/BRCA2* and *ATM*) [8, 14, 76]. Poly (ADP-ribose) polymerase (PARP) inhibitors target a crucial component of the base excision repair pathway appear to be effective against tumors with homologous recombination repair deficiencies. For example, a maintenance regimen with PARP inhibitor olaparib improves PFS in patients with germline *BRCA1/2*-mutated pancreas carcinoma [77]. According to Bang and colleagues [78], a combination of olaparib and paclitaxel improved OS (13.1 vs 8.3 months) in patients with advanced GAC. OS benefit was greater in patients with tumor lacking ATM mutations, but the difference was not statistically significant. These results were not confirmed by the phase III GOLD trial in selected Asian patients with advanced GAC [79], but the study may have been limited by the very small number of ATM-low participants [80]. Despite the contrasting results, the use of PARP inhibitors in GAC is currently under study. For example, the NCT03427814 trial is now evaluating the efficacy of pamiparib-including maintenance therapy in patients with advanced-stage GAC, while trials NCT03008278, NCT04209686 and NCT02678182 are investigating the combination of PARP inhibitors with angiogenesis inhibitors and ICIs [20].

### EGFR-targeted therapies

EGFR amplification and EGFR overexpression are reported in approximately 5–10% of GACs and are considered markers of poor prognosis [81, 82]. Anti-EGFR therapies have already been investigated in GAC, but with disheartening results. Phase III trials EXPAND [83] and REAL-3 [84] have failed to demonstrate significant survival benefits when adding an anti-EGFR antibody to first-line chemotherapy in unselected patients with metastatic GAC. On the other hand, it has been suggested that anti-EGFR therapies have significant activity against tumors with high levels of EGFR expression, supporting the need for a better characterization of the EGFR status for future clinical trials [20, 84, 85]. However, Smyth and colleagues [86] report a particularly poor prognosis for *EGFR*-amplified GACs treated with EGFR inhibitors in combination with chemotherapy, probably due to an antagonistic effect between anti-EGFR agents and the chemotherapeutic drug epirubicin. Other mechanisms of resistance to anti-EGFR therapies are co-amplification of *HER2*, *NRAS*, *KRAS*, *MYC*, or *CCNE1*, and mutation of *KRAS* or *GNAS* [84, 86].

### FGFR-targeted therapies

*FGFR2* amplifications occur in 5–10% of GACs and have been associated with poor prognosis and with CIN and GS molecular subtypes [20, 87, 88]. AZD4547 represents one of the first selective pan-FGFR TKIs developed. Despite encouraging preclinical results [89], phase II trial (SHINE) failed to demonstrate improved PFS with AZD4547 compared with paclitaxel in the second-line treatment of patients with metastatic GACs harboring *FGFR2* polysomy or FISH-detected amplifications [90].

However, intratumor heterogeneity of *FGFR2* amplification and poor concordance between *FGFR2* amplification/polysomy and expression may hamper the case selection of clinical trials [90].

Others FGFR-targeted therapies are currently under investigation. The FGFR1-4 irreversible inhibitor futibatinib is being tested in a phase II trial involving patients with advanced-stage solid tumors harboring FGFR alterations (NCT02052778) [91]. Bemarituzumab is a monoclonal antibody against the FGFR2b splice variant that is frequently overexpressed in FGFR2-amplified GACs, and its use has been investigated with promising results [20]. Results from phase II FIGHT trials suggest that the addition of bemarituzumab to mFOLFOX6 led to clinically meaningful and statistically significant improvements in PFS (9.5 vs 7.4 months), OS (not reached vs 12.9 months) and ORR (47% vs 33%) in patients with previously untreated, FGFR2b-overexpressing advanced-stage GACs [92]. Phase III FIGHT trial is currently ongoing [93].

### MET-targeted therapies

Alteration of the MET/ hepatocyte growth factor (HGF) pathway has been associated with more aggressive disease and poorer prognosis in different gastrointestinal neoplasms [94].

A combination of mFOLFOX6 plus HGF-signaling inhibitor onartuzumab has been investigated in a METGastric trial [95], without any significant improvement in OS, PFS or ORR. Moreover, the RILOMET-1 trial [96] failed to prove the significant clinical benefits of c-MET-signaling inhibitor rilotumumab in patients with MET-positive GACs. Despite these results, some studies suggest that MET TKIs have significant antitumor activity in patients with a high level of *MET* amplification [97, 98]. The potential efficacy of anti-MET therapies is probably undermined by the development of acquired resistance. According to Frigault and colleagues [99], *MET* D1228 V/N/H and Y1230C mutations or high copy number *MET* gene amplifications are responsible for the acquisition of resistance to savolitinib in patients with *MET*-amplified GAC.

### Other possible therapeutic scenarios

Anti-angiogenic therapies have been investigated in GAC, with contrasting results.

The addition of anti-VEGFA antibody bevacizumab to chemotherapy as first-line treatment has not been associated with an improvement in OS [100, 101]. On the other hand, studies evaluating the effectiveness of ramucirumab in the second-line setting have provided some interesting results. In the phase III RAINBOW trial [102], a combination of ramucirumab with paclitaxel has been reported to increase OS (9.6 vs 7.4 months), when compared with placebo plus paclitaxel. Moreover, according to Fuchs and colleagues [103], ramucirumab monotherapy significantly improves OS (5.2 vs 3.8 months) in GAC patients. The VEGFR-2 TKI apatinib has been associated with increased overall and PFS compared to placebo in patients with advanced GAC refractory to at least two lines of chemotherapy [104]. However, these results have not been confirmed in the phase III ANGEL trial [105].

Regorafenib is a multi-kinase inhibitor that also targets angiogenic receptor tyrosine kinases and is currently used in colorectal cancer and GIST patients [106]. In the phase II INTEGRATE trial [107], regorafenib has been associated with longer PFS (2.6 vs 0.9 months) in previously treated GC compared to placebo. Phase III INTEGRATE trial (NCT02773524) is currently ongoing [20].

Overexpression of Matrix Metallo Proteinase-9 (MMP-9) in GAC has been associated with poor prognosis [108]. The addition of MMP-9 inhibitor andecaliximab to FOLFOX presented encouraging antitumoral activity against GAC in the phase I/Ib trial [109], but those results have not been confirmed in subsequent studies (GAMMA-1) [110].

Finally, a combination of pembrolizumab and a modulator of the Wnt and PI3KeAKT signaling pathways Dickkopf-1 (DKK1) has been tested in GC patients, with promising results [111].

## Liquid biopsy

Intratumoral and intertumoral heterogeneity may fuel therapeutic resistance and treatment failure in cancer patients. Longitudinal profiling of serial blood samples may help to identify and characterize acquired drug resistance and track cancer evolution over time. In this context, the application of liquid biopsy in the clinic may be a powerful tool to guide therapeutic-decision progress in case of genomically discordant tissue samples or whether biopsy sampling cannot be performed. In a study by Pectasides and colleagues, genomic tissue profiling identified discordance of common alterations (*HER2, MYC, CCND1, EGFR*) between primary tumor and metastasis in 32% of patients. However, in most discordant cases, the analysis of circulating-free DNA (cfDNA) was concordant with the metastatic tissue, suggesting the potential for cfDNA profiling to more effectively guide the therapeutic choice [112]. In a cohort of 42 gastrointestinal cancer patients with acquired resistance to targeted therapy, post-progression cfDNA profiling was found to identify more accurately resistance alterations when compared to tissue analysis [113]. A recent study by Nakamura and colleagues [114] compared trial enrollment using circulating tumor DNA (ctDNA) sequencing to enrollment using tumor tissue sequencing in 1,687 patients with advanced gastrointestinal cancer. According to the results, ctDNA profiling significantly shortened the screening duration and improved the trial enrollment rate compared to tissue genotyping, thus representing a possible source of advance and innovation in the delivery of precision oncology.

In the metastatic setting, the levels of cfDNA and ctDNA positively correlate with tumor burden. For this reason, liquid biopsy has emerged to predict recurrence or relapse in gastric cancer following surgical resection [115].

## Conclusions

GAC still remains an aggressive and deadly neoplasm. The great efforts made to develop novel effective molecular targeted therapies have not been followed by completely satisfying results. A lesson that can be learned from unsuccessful clinical trials is that adequate patients’ selection should always be a priority in both clinical studies and daily practice. Cornerstone of this process is a wise use of predictive biomarkers, which are necessary tools to identify those patients that will benefit from targeted therapy.

On the other hand, the use of biomarkers in clinics and in clinical research settings has several limitations. In fact, a single biomarker is not representative of the biologic complexity of the neoplasm and of the high level of genomic and molecular interpatient heterogeneity.

In the era of biomarker-driven oncology, the pathologist plays a central role in the process of therapeutic choice, which means that they must correctly perform the evaluation and avoid possible pitfalls. For this reason, in the context of gastroesophageal malignancies, biomarker assessment should always be performed by an expert and dedicated gastrointestinal pathologist.

The effectiveness of targeted therapies may be negatively affected by the high levels of spatial and temporal intratumor heterogeneity found in GAC. It is, therefore, crucial to perform adequate sampling that is representative of the entire neoplasm. However, heterogeneity is not a static concept since new molecular alterations may appear with time. Moreover, therapies can induce a selective pressure that gives proliferative advantages to resistant subclones, changing the proportion of different cellular populations that made up the primitive tumor. This implies that the initial biomarkers’ status may not be considered definitive, and re-assessment is highly recommended in recurrent and metastatic lesions. In this setting, a liquid biopsy may represent a powerful tool to guide the therapeutical decision process.
